# Antimicrobial Hydroxyethyl-Cellulose-Based Composite Films with Zinc Oxide and Mesoporous Silica Loaded with Cinnamon Essential Oil

**DOI:** 10.3390/pharmaceutics16091225

**Published:** 2024-09-19

**Authors:** Ludmila Motelica, Denisa Ficai, Gabriela Petrisor, Ovidiu-Cristian Oprea, Roxana-Doina Trușcǎ, Anton Ficai, Ecaterina Andronescu, Ariana Hudita, Alina Maria Holban

**Affiliations:** 1 Faculty of Chemical Engineering and Biotechnologies, National University of Science and Technology POLITEHNICA Bucharest, 1-7 Gh. Polizu, 011061 Bucharest, Romania; ludmila.motelica@upb.ro (L.M.); gabriela.petrisor06@yahoo.com (G.P.);; 2 National Center of Micro and Nanomaterials, National University of Science and Technology POLITEHNICA Bucharest, Splaiul Independentei 313, 060042 Bucharest, Romania; alina.m.holban@bio.unibuc.ro; 3 Academy of Romanian Scientists, 3 Ilfov St., 050044 Bucharest, Romania; 4 Faculty of Biology, University of Bucharest, 077206 Bucharest, Romania; ariana.hudita@bio.unibuc.ro

**Keywords:** hydroxyethyl cellulose, ZnO, biodegradable packaging, MCM-41, cinnamon essential oil, antibacterial, synergy

## Abstract

**Background**: Cellulose derivatives are gaining much attention in medical research due to their excellent properties such as biocompatibility, hydrophilicity, non-toxicity, sustainability, and low cost. Unfortunately, cellulose does not exhibit antimicrobial activity. However, derivatives like hydroxyethyl cellulose represent a proper matrix to incorporate antimicrobial agents with beneficial therapeutic effects. **Methods**: Combining more antimicrobial agents into a single composite material can induce stronger antibacterial activity by synergism. **Results**: Therefore, we have obtained a hydroxyethyl-cellulose-based material loaded with zinc oxide nanoparticles and cinnamon essential oil as the antimicrobial agents. The cinnamon essential oil was loaded in mesoporous silica particles to control its release. **Conclusions**: The composite films demonstrated high antibacterial activity against *Staphylococcus aureus* and *Escherichia coli* strains, impairing the bacterial cells’ viability and biofilm development. Such antimicrobial films can be used in various biomedical applications such as topical dressings or as packaging for the food industry.

## 1. Introduction

Extensive and usually uncontrolled microbial development on the surface of foodstuff leads to spoilage and limits shelf life [1]. Despite many research efforts, the industry is still slow to adopt antimicrobial packaging. The main polymers used by the food industry as packaging are petroleum-based materials [2] and cellulose [3].

Cellulose is the most abundant natural polymer used as packaging but unfortunately it has no intrinsic antimicrobial activity [4]. Among the cellulose derivatives, the literature concerning hydroxyethyl cellulose (HEC) reports a weak, but detectable antibacterial activity, that some authors assign to the citric acid used for cross-linking [5]; however, citric acid is not always reported as a component [6,7,8,9,10]. HEC is a cellulosic ether derivative, approved by the U.S. Food and Drug Administration (FDA), used in cosmetics and the food industry [11]. HEC-based materials are developed as wound dressings [12] but also as biodegradable packaging [13]. Characteristic for HEC is the large number of accessible –OH moieties that can interact easily with other functional/polar compounds or particles.

As antimicrobial resistance becomes a stringent problem of modern society, the quest for new strategies to combat bacterial infections is attracting more scientists. One of the strategies to obtain strong antibacterial activity is to synergistically combine multiple antimicrobial agents in the same material [1]. This strategy leads to a lower required quantity of antimicrobials for a certain effect, but can lead to a stronger and better antibacterial material against a wider spectrum of microorganisms [14]. Therefore, adding ZnO nanoparticles (NPs) and cinnamon essential oil to the HEC should lead to an improvement of the antibacterial potency of the composite film.

Natural plant extracts and essential oils (EOs) are also a major research topic, due to their multiple health benefits, which include antioxidant or antimicrobial activities [15,16]. Usually, one or two of the main components from an essential oil are responsible for the antibacterial activity [17]. Nevertheless, minor components can contribute to the overall results by sensitizing the microorganisms or by synergism. Previous reports indicate a strong antimicrobial activity for cinnamon essential oil (CEO) [17,18]. Such strong antibacterial and antifungal natural extracts are good candidates for innovative packaging or wound dressing materials. *E*-cinnamaldehyde is the main component of CEO (72%) and at the same time is the principal antimicrobial agent. The other important minor components are β-caryophyllene (6.5%), eugenol (4.5%), eucalyptol (5.5%), and linalool (7%) [17]. As many of the components are volatile, a sustained antibacterial activity can be ensured if the CEO is encapsulated or loaded in a proper matrix [19,20]. Such delivery systems can ensure a suitable release profile for various applications. Volatile components can be retained for longer time due to interactions with encapsulation systems or nanoparticle surfaces, and this will lead to a slower release over time for CEO components, providing a long-lasting antimicrobial activity [21,22].

Zinc oxide is classified as safe by the Food and Drug Administration, while the studies of the European Food Safety Authority (EFSA) concluded that ZnO does not migrate in nanoform, and recommend a limit between 5 and 25 mg ZnO/kg in food (article 10/2011 from the Plastics Regulation of Commission Regulation) [23]. The antibacterial and antifungal activities of ZnO NPs are well known in the literature, albeit in large limits due to various synthesis methods [24,25,26]. Strong antibacterial activity was reported by us for ZnO NPs obtained by the forced solvolysis method due to shape, size, and surface defects that increase their potency [27,28]. ZnO generates high quantities of reactive oxygen species (ROS) as indicated by its strong photocatalytic activity [29,30]. The ROS generation is one of the main mechanisms by which ZnO becomes toxic for microorganisms. However, alternative killing pathways are mentioned by the literature, based on the antimicrobial activity of ZnO NPs in the absence of light. Mechanical damage to the cellular membrane, like puncture or rupture, represents an alternative mechanism [28], but Zn^2+^ ions released after nanoparticles’ internalization can also disrupt the metabolic pathways by binding proteins, DNA, enzymes, or other essential molecules. Therefore, ZnO NPs are considered as an alternative microbicidal agent in various domains, like water treatment, topical ointments, or food packaging [29,31,32,33].

Combining the antimicrobial activities of ZnO and CEO to obtain a strong synergic response is a good strategy for developing novel composite materials [34,35,36]. Nevertheless, the amount of essential oil that can be loaded on the surface of ZnO is limited due to its surface area and its non-porous nature in general. A specific surface area of ~34 m^2^/g and pore volume values of 0.15 cm^3^/g for ZnO nanoplatelets are reported in [37]. A higher specific surface area of 43 m^2^/g but lower pore volume of 0.089 cm^3^/g are reported for ZnO irregular nanoparticles in [38]. By contrast, when the synthesis is mediated by plant extracts, a specific surface area of ~23 m^2^/g is reported for nanorods in [39] and only ~13 m^2^/g for 40 nm nanoparticles in [40]. In the case of synthesis made in ionic liquid, an area of 29 m^2^/g is reported [40]. In a study of precursor influence on the properties of ZnO NPs, the largest specific surface area is 11 m^2^/g, while pore volume is 0.07 cm^3^/g [41]. It should be noted that the pores for ZnO are in fact inter-particles spaces, with each nanostructure being compact and without identifiable pores. A sensible higher specific surface area of ~137 m^2^/g is reported for mesoporous ZnO structures which are assembled in a complicated synthesis from ZnO nanocrystals of 5 nm [42].

Mobile Composition of Matter family with a hexagonal pore structure (MCM-41) is a type of mesoporous silica material with a high surface area (1365 m^2^/g) and a high volume of pores (0.783 cm^3^/g) [43]. The pores are arranged in a uniaxial fashion, which leads to the unidirectional release of the loaded active components [44]. MCM-41 can be loaded with sizably larger amounts of natural extracts and act both as a reservoir and stabilizing factor for the loaded compounds [45]. Therefore, some of the previous literature reports the successful loading of mesoporous silica with essential oils [46,47,48].

In the present research, we report for the first time the obtaining, characterization, and antibacterial properties of a cellulose-based composite containing HEC, ZnO NPs, and MCM-41 particles loaded with CEO.

## 2. Materials and Methods

Zinc acetate, Zn(CH_3_COO)_2_·2H_2_O, glycerol (C_3_H_8_O_3_), and cetyltrimethylammonium bromide (CTAB) were obtained from Merck (Amex-Lab SRL, Bucharest, Romania). Ammonia solution (NH_4_OH), tetraethyl orthosilicate (TEOS), cinnamon essential oil (CEO), and n-butanol were purchased from Sigma Aldrich (Redox, Bucharest, Romania), while hydroxyethyl cellulose type H200000YP2 was obtained from Shin-Etsu (Tokyo, Japan).

### 2.1. Preparation of MCM-41 and ZnO Particles

The preparation of ZnO NPs was conducted as presented in [28]. In short, 5.000 g Zn(CH_3_COO)_2_·2H_2_O was added over 50 mL n-butanol in a round-bottom flask and kept under magnetic stirring at reflux temperature. The white powder was separated by centrifugation and washed trice with ethanol, in the end obtaining ZnO NPs.

The preparation of MCM-41 was conducted as presented in [44] by the soft template method. Briefly, we started the synthesis from 20 mL TEOS as the silica source and 5 g CTAB as the template agent. The obtained precipitate was washed and then calcined to 550 °C. The purity of the samples was assessed by FTIR, XRD, and TG-DSC.

### 2.2. Obtaining of the Cellulose-Based Composite Films

A certain amount of hydroxyethyl cellulose (5 g) was dissolved under stirring in 100 mL water and 2 mL glycerol was added as a plasticizer. Separately, the MCM-41 particles were loaded with cinnamon essential oil, by the vacuum-assisted method, as indicated in Figures S1 and S2. Briefly, the MCM-41 was firstly kept under vacuum at ~0.1 bar for 30 min, on ice bath, in a Schlenk flask, followed by the addition by injection of the CEO to be adsorbed into the pores and pressurization of the flask [49].

MCM-41 (0.25 g) was loaded with CEO (1.5 mL) and afterwards mixed with a certain amount of ZnO NPs (Table 1). The loaded particles were added into the HEC solution under vigorous stirring. The obtained solutions were used to fabricate the composite films by the casting method in Petri dishes. The films were dried in an oven for 24 h at 40 °C.

Additionally, intermediary compositions were used to form the C5 and C6 samples that were used to assess the influence of each agent on antibacterial activity. C6 is similar to C2 but with no ZnO, while C5 is similar to C3 but with no MCM-41 or CEO.

### 2.3. Characterization Techniques

#### 2.3.1. Microstructural Analysis

The films’ surface morphology and microstructure were investigated by scanning electron microscopy (SEM) using the QUANTA INSPECT F50 equipment (FEI Company, Eindhoven, The Netherlands) with an energy dispersive X-ray spectrometer (EDS) used to map the distribution of the elements across the samples.

#### 2.3.2. Fourier Transform Infrared Spectroscopy

The presence of certain functional groups and their interactions were investigated by Fourier transform infrared (FTIR) spectroscopy in the domain 400–4000 cm^−1^ with a Nicolet iS50 spectrometer (Thermo Fisher Scientific Inc., Waltham, MA, USA). The spatial distribution of components was assessed by FTIR microscopy with the help of a Nicolet iN10 MX microscope (Thermo Fisher Scientific Inc., Waltham, MA, USA).

#### 2.3.3. Ultraviolet–Visible Spectra Measurements

The ultraviolet–visible spectra (UV–vis) were recorded in the domain 200–900 nm with a JASCO V560 spectrophotometer (JASCO Inc., Easton, PA, USA). The spectrometer was equipped with a 60 mm integrating sphere and a film holder. The opacity values were calculated as presented in [33], as A_600_/*x*, where A_600_ is the absorbance at 600 nm and *x* is the film thickness in mm. A higher opacity value indicates that the film is less transparent.

#### 2.3.4. Photoluminescence Spectra Measurements

The photoluminescence spectrum (PL) was recorded with the help of a Perkin Elmer LS55 spectrometer (Waltham, MA, USA), by using an excitation wavelength of 320 nm, with a 350 nm cut-off filter, a scanning speed of 200 nm·min^−1^, and 10 nm slits.

#### 2.3.5. Thermal Analysis

The thermal stability was followed with a STA 449C F3 system, TG-DSC (thermogravimetry–differential scanning calorimetry) from Netzsch (NETZSCH-Gerätebau GmbH, Selb, Germany), between 20 and 900 °C, in dynamic (50 mL/min) air atmosphere. The evolved gases were transferred through heated transfer lines and analyzed on the fly with the help of a FTIR Tensor 27 from Bruker (Bruker Co., Ettlingen, Germany), equipped with an internal thermostatic gas cell.

#### 2.3.6. Water Vapor Permeability Measurements

The water vapor permeability (WVP) was determined as described in [1] by using glass vials of 12 mm diameter sealed with a sample film. Each vial contained 1.1 g anhydrous CaCl_2_. The vials were placed in a desiccator at T = 298 K and humidity 100%. At determined time intervals, the mass of the vials was measured to determine the amount of water that passed through the films. The WVP was calculated by Equation (1):

(1) $$WVP=\frac{\left(M_{t}-M_{0}\right)}{∆t\times ∆P\times S}\times L$$

where M_t_ and M_0_ are masses of vials at time t and t = 0, L is the film thickness (in m), S represents the area of the film samples (in m^2^), ∆P is the water vapor pressure difference on both sides of the film at 25 °C and a relative humidity gradient of 100% (∆P = 3169 Pa), and ∆t is the time (in seconds).

#### 2.3.7. Film Expansion Profile Measurements

Film expansion determinations were made by using a 4% gelatine solution. Freshly prepared gelatine solution was poured into Petri dishes and left for 24 h at 25 °C. Circular samples of each film were then placed on top of the gel. The increase of the diameter for each sample was measured at certain time intervals. The film expansion (%) was calculated with Equation (2):

(2) $$Filmexpansion(\%)=\frac{\left(D_{t}-D_{0}\right)}{D_{0}}\times 100$$

where D_t_ and D_0_ are the samples’ diameters at time t and t = 0.

#### 2.3.8. In Vitro Release Study

The release profile for the principal component of CEO, cinnamaldehyde, was measured with a JASCO V560 spectrophotometer (JASCO Inc., Easton, PA, USA) at 288 nm. Briefly, disc samples of 6 mm and ~0.0200 g were placed in 50 mL phosphate-buffered saline (PBS) solution and kept under stirring. At certain time intervals, the absorbance at 288 nm was measured for the samples taken.

### 2.4. Antibacterial Assay

The antibacterial activity was evaluated against one Gram-positive (*Staphylococcus aureus* ATCC 25923) and one Gram-negative (*Escherichia coli* ATCC 25922) opportunistic pathogen, recognized for their impact in wound infections. The strains were acquired from ATCC and maintained as glycerol stocks at −80 °C until use.

For the antibacterial qualitative assessment (growth inhibition), we utilized an adapted diffusion assay, respecting the general rules exposed in the CLSI 2023. As a standardized inoculum, we used a 0.5 McFarland bacterial suspension, corresponding to 1.5 × 10^8^ CFU (colony forming units)/mL, prepared in 0.9% NaCl sterile saline. The previously prepared solution was used to inoculate Petri dishes containing Mueller–Hinton agar. Samples cut as 6 mm diameter discs were prepared from each film. Before use, the discs were sterilized by UV irradiation for 30 min. Sample discs were aseptically placed on the inoculated Petri dishes and these were incubated for 24 h at 37 °C. After incubation, the diameter of growth inhibition developed around each sample disc was measured (in mm).

For the bacterial viability analysis, UV-sterilized samples were aseptically placed in a well of a sterile 24-well plate. Over each specimen, 10 µL of 10^7^ CFU/mL microbial suspensions prepared in PBS (phosphate-buffered saline) were added. The prepared plates were incubated at 37 °C for 1, 6, or 24 h. The reduction of bacterial cells’ viability was evaluated by using serial dilution of the suspensions after contact with materials and plating such dilutions on Mueller–Hinton agar, which was followed by their incubation at 37 °C for 20 h. After the incubation, the viable counts were expressed as number of CFU/mL.

The antibiofilm efficiency was determined by incubating the material specimens (sterilized by previous UV exposure, 6 mm in diameter) in sterile 24-well plates containing 1 mL nutritive broth, followed by the inoculation of 10 μL bacterial suspension of 0.5 McFarland standard density. Afterwards, the plates were incubated at 37 °C for 24 h. After the incubation time, the unattached microbial cells were discarded by washing the samples with 1 mL of sterile saline solution. The samples were transferred to sterile plates containing fresh nutritive broth to allow biofilm development for 24 h. In the end, the samples were gently washed with 1 mL of sterile saline and transferred after in centrifuge tubes containing 1 mL sterile saline solution. In order to detach the biofilm cells, the samples were vortexed for 20 s and exposed to ultrasounds for 10 s. After this, serial 10-fold dilutions were obtained and then inoculated on Mueller–Hinton agar to evaluate the viable colony formation, expressed as CFU/mL. All experiments were performed in triplicates. The data from the antibacterial assay were processed with the GraphPad Prism v9 software (San Diego, CA, USA), using the one-way ANOVA statistical tool.

### 2.5. Biocompatibility Assessment

The biocompatibility of the synthesized materials (C1 to C4) was assessed using the human keratinocytes HaCaT cell line. Cells were cultured in Dulbecco’s Modified Eagle Medium (DMEM, Sigma-Aldrich, Steinheim, Germany) supplemented with 10% fetal bovine serum (FBS, Gibco, Thermo Fisher Scientific, Waltham, MA, USA) and 1% penicillin–streptomycin antibiotic mixture (ABAM, Sigma-Aldrich). Standard cell culture conditions of 37 °C, 95% humidity, and 5% CO_2_ were maintained throughout the experiments. Before cell seeding, the materials were sterilized via UV exposure for 20 min on both sides and transferred under aseptic conditions to 48-well sterile cell culture plates. HaCaT cells were seeded on the material surfaces in droplets at an initial cell density of 1.5 × 10^4^ cells/cm^2^ and immersed in complete DMEM culture medium after 1h. The cells were cultured for 72 h under standard conditions. The C1 formulation was used as the experimental control for in vitro investigations.

Cell viability and proliferation were evaluated using the MTT assay at 24 h and 72 h post-contact with the materials. Briefly, the cell culture medium was discarded and replaced with a fresh MTT solution prepared in FBS-free medium at a final concentration of 1 mg/mL MTT ([3-(4,5-dimethylthiazol-2-yl)-2,5-diphenyltetrazolium]) (Sigma-Aldrich). After 4-h of incubation in standard cell culture conditions, the MTT solution was discarded and the resulting formazan crystals were dissolved in isopropanol. The absorbance of the obtained solutions was measured at 550 nm using the FlexStation III multimodal plate reader (Molecular Devices, San Jose, CA, USA). The results were expressed as percentage cell viability relative to the control, which was considered 100% viability at 24 h. Lactate dehydrogenase (LDH) release was measured to evaluate the impact of material–cell contact on membrane integrity. After 24 h and 72 h of contact, cell culture supernatants were collected and processed using the TOX-7 kit (Sigma-Aldrich) according to the manufacturer’s instructions. After 30 min of incubation, the reaction was stopped with 1N HCl, and absorbance was measured at 490 nm.

## 3. Results and Discussion

### 3.1. Scanning Electron Microscopy (SEM) Characterisation

**Figure 1:**
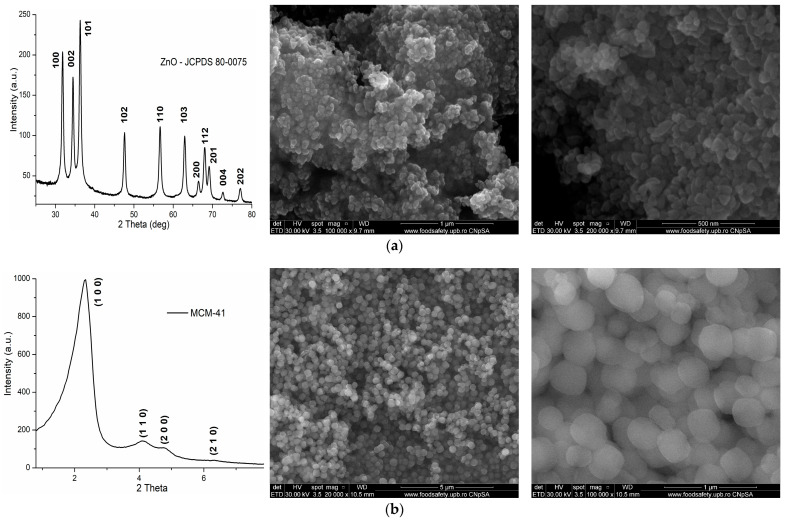
The XRD and SEM micrographs for the ZnO (**a**) and MCM-41 (**b**) particles.

The SEM micrographs for ZnO and MCM-41 particles are presented in Figure 1, together with the XRD patterns that identify the pure crystalline phase as ZnO and ordered mesoporous silica, MCM-41 type.

The ZnO NPs are polyhedral, with uniform size and tendency to form soft agglomerates. The medium measured size of ZnO NPs is similar to the calculated crystallite size, ~31 nm (Figure 1a). The XRD pattern is indexed to the hexagonal zincite JCPDS 80-0075 [50].

The MCM-41 particles have a spherical morphology, with a diameter between 150 and 400 nm with no tendency to form an agglomerate (Figure 1b). The XRD pattern shows four characteristic Bragg reflections (100), (110), (200), and (210) [51,52]. The separate peaks are a good indicator of the increased order porosity in the MCM-41 particles [53].

**Figure 2:**
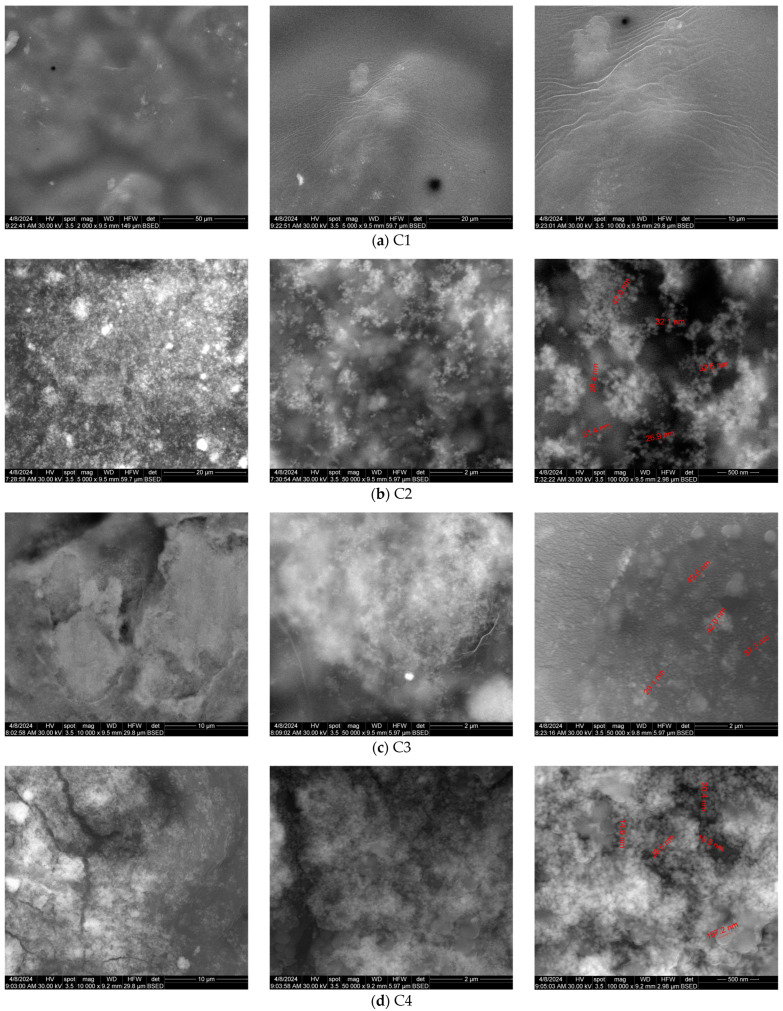
The SEM micrographs for C1 (cellulose film) (**a**), C2 (**b**), C3 (**c**), and C4 (**d**) composite films (with red numbers indicating the size of the particles).

The morphology of the cellulose-based composite films is presented in Figure 2. The micrographs are recorded in back scattering electron diffraction mode to visualize the particle distribution in the samples. The C1 sample composed only from HEC has a neat surface with minimum irregularities, generated by the drying pattern. The composite samples have a more heterogeneous structure, with inorganic particles distributed on the surface and across the film thickness. At low magnification, the presence of some CEO micro-droplets in the films’ structure is noticeable, with a uniform distribution.

In all three samples, C2–C4, the MCM-41 particles are visible as larger, less distinct spheres, surrounded by smaller, clearer ZnO nanoparticles (due to the differences between the atomic numbers of Si and Zn). The distribution of both kinds of particles is quite uniform across the films, indicating a good dispersion into the cellulose matrix (Figure 2).

**Figure 3:**
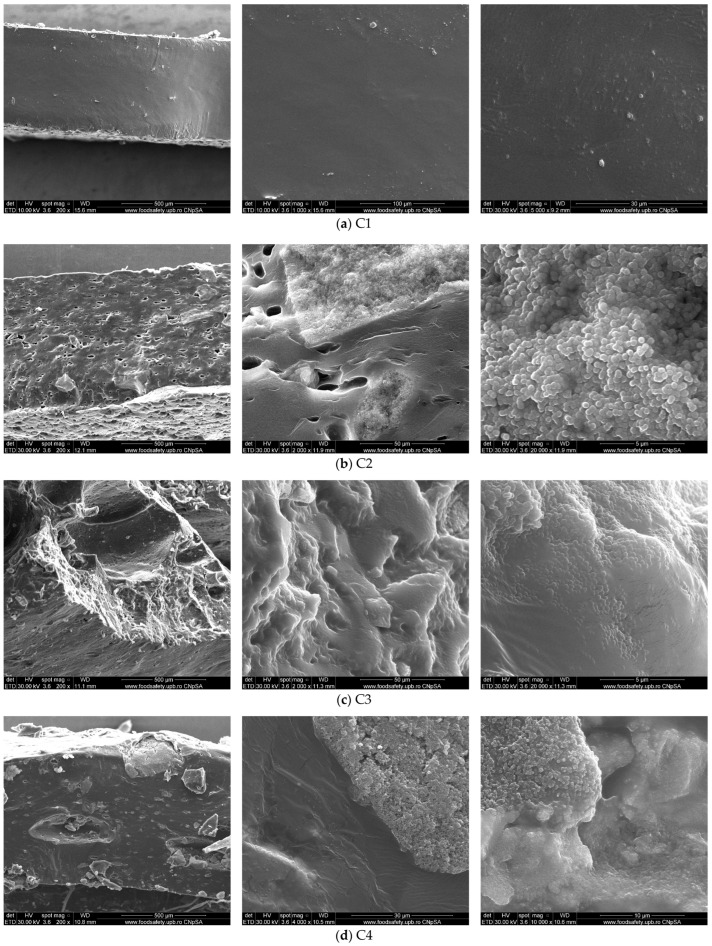
The SEM micrographs for the cryo-fractured cross-sections of C1 (cellulose film) (**a**), C2 (**b**), C3 (**c**), and C4 (**d**) composite films.

The cross-sections of the cryo-fractured film samples are presented in Figure 3. 

In order to gather the general aspect of the fracture, the micrographs are presented at low magnification (200×). In the next micrographs, at higher magnification (up to 20,000×), we noticed the details of the surface and the presence of some particle agglomerations embedded into the HEC matrix, but also the micropores generated inside the film structure by the presence of CEO.

**Figure 4:**
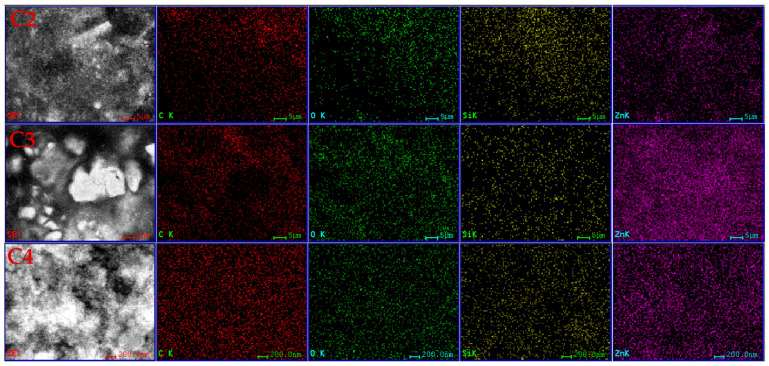
The EDS elemental maps for C2, C3, and C4 cellulose-based composite films.

The analysis (EDS) showed a sharp picture of the homogeneity dispersion degree (Figure 4) for the inorganic particles. It can be noticed that the elemental maps indicate a good dispersion of both MCM-41 (Si yellow map) and ZnO (Zn purple map), without noticeable agglomerations in the overall film structure.

### 3.2. FTIR Spectroscopy and Microscopy

**Figure 5:**
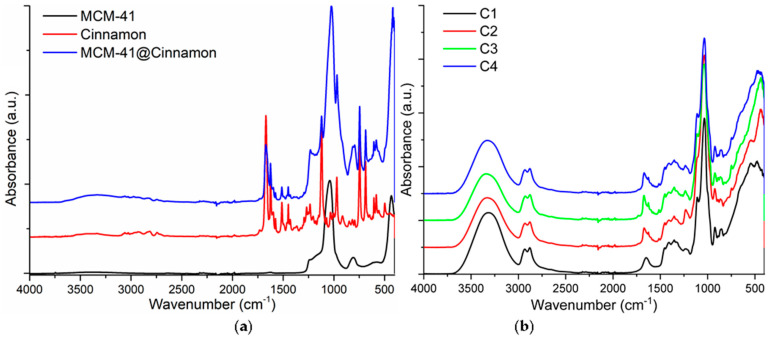
FTIR spectra for cinnamon essential oil, MCM-41 particles, and MCM-41 particles loaded with cinnamon essential oil (**a**); C1 (cellulose film), C2, C3, and C4 cellulose-based composite films (**b**).

The FTIR spectra of CEO, MCM-41, and MCM-41 loaded with CEO are presented in Figure 5a, while the FTIR spectra for cellulose-based films are presented in Figure 5b.

The CEO FTIR spectrum presents the characteristic absorption band (weak) from 1730 cm^−1^, assigned the aldehyde of a saturated compound, and peaks at 1625 and 1670 cm^−1^, originating from the stretching vibration of a C=O from cinnamaldehyde. The lesser peak from 1513 cm^−1^ is assigned to C=C from the aromatic rings, while the one from 1450 cm^−1^ is assigned to the C-OH bending vibration. The weak peak from 2750 cm^−1^ belongs to the C-H stretching vibration in the aldehyde moiety. The strong absorption peak from 1121 cm^−1^ is attributed to aromatic ester C-O-C symmetric vibration from eugenol, while the 750 and 688 cm^−1^ peaks belong to benzene rings and alkenes, respectively [54]. All these characteristic peaks are easily identifiable in MCM-41 loaded with the CEO sample indicating a successful encapsulation. The MCM-41 has a simpler FTIR spectrum, with peaks at 1058, 808, and 440 cm^−1^ corresponding to asymmetric Si-O-Si vibration, symmetric, and bending Si-OH vibrations, respectively [55].

The cellulose-based films present strong absorption bands at 3316 cm^−1^ assigned to the O-H stretching vibration, 2936 and 2879 cm^−1^ attributed to the C-H asymmetric and symmetric stretching vibrations from CH_2_ and CH_3_, 1648 cm^−1^ from the glucose ring, and 1035 cm^−1^ from the C-O bond [11,56]. The presence of CEO in the C2–C4 composite samples is confirmed by the small peaks at 1625, 1513, and 750 cm^−1^. The presence of ZnO is confirmed by the strong peak at 436 cm^−1^ attributed to the Zn-O bond stretching vibration [57].

**Figure 6:**
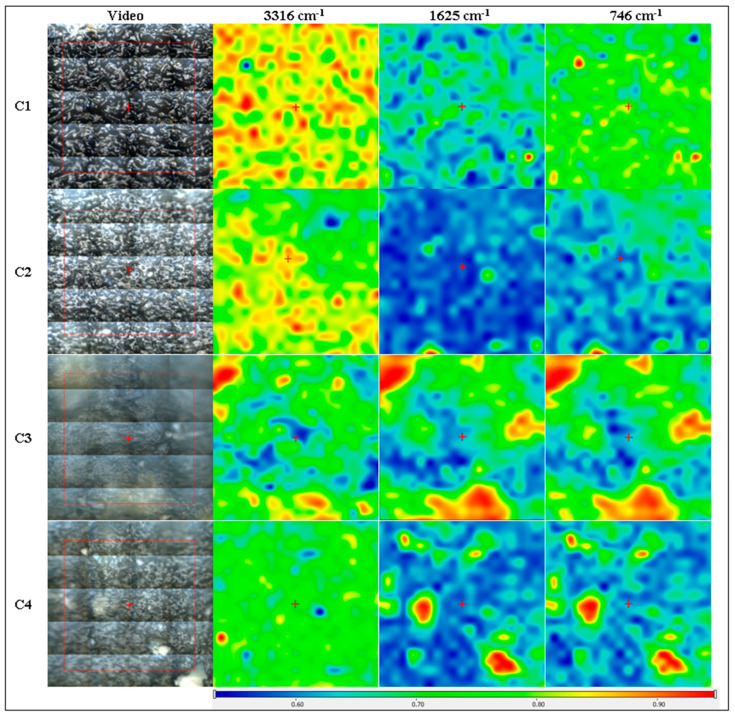
FTIR microscopy maps for C1 (cellulose film), C2, C3, and C4 cellulose-based composite films at 3316, 1625, and 746 cm^−1^; red areas indicate the highest absorbance, while blue areas correspond to the lowest absorbance.

The FTIR microscopy maps for cellulose-based films C1–C4 are presented in Figure 6 at three specific wavenumbers, 3316 cm^−1^ for O-H stretching vibration in HEC, 1625 and 746 cm^−1^ for cinnamaldehyde and aromatic compounds from CEO. The sample C1 that contains only HEC present with the most uniform maps. For C2–C4, there are some observable heterogeneity degrees, but these are at the micrometer level [58,59]. As indicated by the SEM micrographs, these heterogeneities are generated by small agglomerations of particles (especially MCM-41).

### 3.3. UV–Vis Spectroscopy

The composite films C2–C4 are colored while the C1 is transparent. The yellowish tint of the composite films is due to the interactions between the cellulose back-bone and inorganic particles, ZnO and SiO_2_, and due to the presence of CEO [60]. The pure HEC film, C1, is transparent in the visible domain, but exhibits a strong absorption band in the UV region, with a maximum at 338 nm. The composite films C2–C4 present with two absorption maxima in the UV region, around 262–272 nm due to the presence of mesoporous silica particles and at 361–382 nm due to presence of ZnO NPs. Both SiO_2_ and ZnO are semiconductors, and as such, they have a valence band (VB) and a conduction band (CB) separated by a forbidden band [61]. The absorption peaks from the UV domain are directly related with the energy separation between VB and CB (the magnitude of the forbidden band) and electron jumps between VB and CB. Unfortunately, a misconception that assigns this absorption band to the surface plasmon resonance (SPR) is gaining traction in the literature [62,63]. This problem is generated by indiscriminately treating oxide NPs like metallic NPs [63].

**Figure 7:**
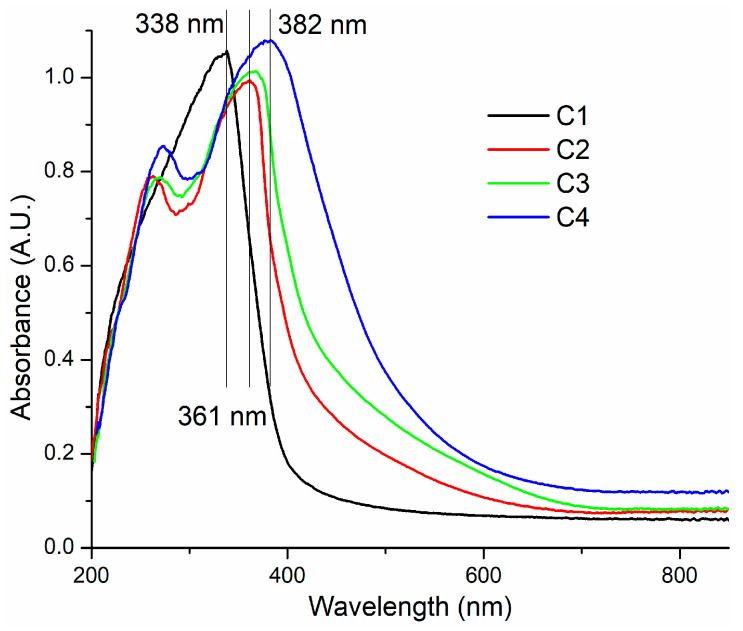
UV–vis spectra for C1 (cellulose film), C2, C3, and C4 cellulose-based composite films.

For both absorption maxima, a bathochromic shift can be observed in the series C2 to C4 indicating the presence of additional energy levels inside the forbidden band, induced by the interactions with the organic components (HEC and CEO). As only the ZnO quantity increases from C2 to C4, it is normal that the corresponding peak suffer a higher shift (~20 nm), as seen in Figure 7. Most probably, the interactions between ZnO and HEC are making the later more susceptible to generate some additional interactions with SiO_2_ particles (~10 nm peak shift). These surface interactions between oxide particles and HEC are sufficient to modify the individual energy of the specific bonding/antibonding orbitals and therefore induce additional electronic levels between VB and CB.

The absorption tail from the 382 nm peak extends in visible domain between 400 and 600 nm and is responsible for the yellowish tint of the composite films. As this tail is missing for the C1 film, this is transparent and colorless. Nevertheless, the high absorbance in the UV domain means that the films are blocking the high-energy photons that are responsible for the initiation of oxidation reactions of lipids, vitamins, and other less stable nutrients, making such films suitable as packaging [64].

### 3.4. Photoluminescence Spectroscopy

**Figure 8:**
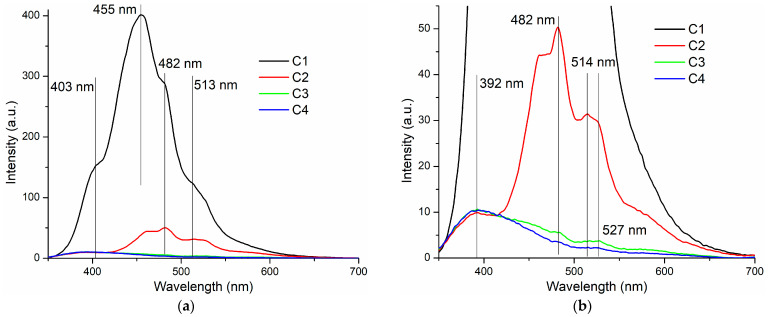
The fluorescence emission spectra for C1 (cellulose film), C2, C3, and C4 cellulose-based composite films (**a**); detailed zoom-in (**b**).

The photoluminescence (fluorescence) spectra of the HEC (C1) and composite films (C2–C4) are presented in Figure 8. 

Hydroxyethyl cellulose, like any cellulose-based material, presents with a strong blue fluorescent emission [65,66,67]. The cellulose fluorescence is quenched when embedded particles are interacting with the polymer. This indicates that the moieties responsible for the fluorescent emission are chemically interacting with the particles’ surface, meaning the composite is not a simple physical mixture.

At the same time, the fluorescence spectrum of ZnO is quenched as the concentration of NPs increases; the only emission band that remains unchanged is the 392 nm one, assigned to the near band edge emission (NBE). This NBE is generated by the free exciton recombination. The visible part of the ZnO fluorescence spectrum is generated by surface defects. These violet–blue–green bands decrease strongly in intensity, indicating the presence of surface interactions between ZnO NPs and HEC.

### 3.5. Thermal Analysis TG-DSC

**Figure 9:**
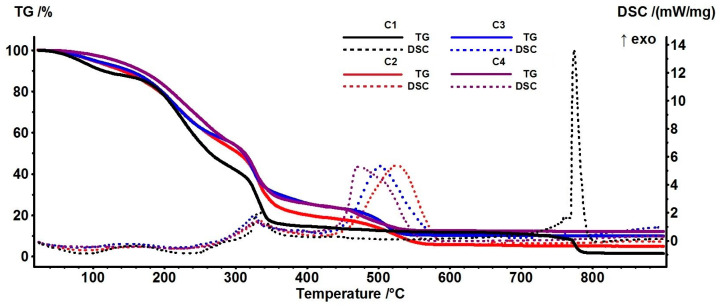
TG-DSC curves for C1 (cellulose film), C2, C3, and C4 cellulose-based composite films.

The thermal analysis TG and DSC curves for all four films are presented in Figure 9. While all the films are losing water up to 150 °C by the dehydrating process of HEC, the composite films are more stable as the ZnO quantity increases, a feature previously reported for other polymer-based composites with ZnO [64].

The presence of inorganic fillers increases the thermal stability of polymers, as the nanoparticles act as cross-linking points for the polymer chains, stabilizing them, and diminishing the external available energy for fragmentation [2].

The principal experimental data from the thermal analysis are presented in Table 2. 

The fragmentation of the HEC backbone take place between 150 and 300 °C, while after 300 °C, the oxidation reactions are dominant [56]. The exothermic peak at ~500 °C is due to the burning of carbonaceous mass. As expected, the residual mass increases from C2 to C4 as the quantity of ZnO NPs is higher.

**Figure 10:**
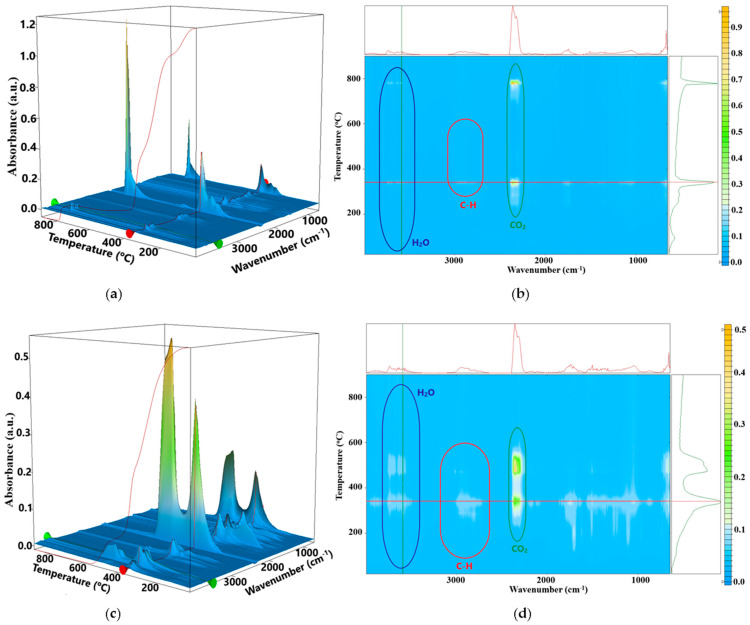
The FTIR 3D diagram for the C1—control (**a**) and C4 sample (**c**); their 2D projections in the temperature/wavenumber plane C1 (**b**) and C4 (**d**), respectively; on top of each 2D projection is the FTIR spectrum at the temperature of 341 °C; at the right side of each 2D projection is the evolving trace for the wavenumber 3582 cm^−1^ assigned to the O-H vibration from water.

The FTIR diagrams of the evolved gases (Figure 10) can give additional information on the degradation pathways [60]. The hydrocarbon fragments are eliminated at a lower temperature for the C4 sample when compared with C1. This is due to the presence of CEO in the C4 composition, with the volatile compounds (especially cinnamaldehyde) being eliminated as low as 100 °C.

Figure S3a presents the FTIR spectra of evolved gases at 144, 233, and 313 °C. The 2733 cm^−1^ C-H stretching vibration, characteristic for aldehydes, is clearly visible at low temperatures, indicating that cinnamaldehyde is eliminated from the sample. Water and CO_2_ are eliminated together in two degradation steps at ~340 and ~500 °C, identifying these as oxidation processes. Nevertheless, water is eliminated also under 200 °C, without the presence of CO_2_, confirming the dehydration process (Figure S3b).

### 3.6. Water Vapor Permeability

Water vapor permeability is an important characteristic for packaging films or wound dressing materials. A low WVP value is essential to keep a moist environment around a wound. For packaging applications, the ability to block water loss from the food leads to the capacity of preserving a stable environment around it and therefore prolongs shelf life. The values calculated for the C1 to C4 samples are presented in Table 3.

The decrease in the WVP value between the samples C1 and C2 can be explained by the presence of SiO_2_ and ZnO NPs, acting as cross-linking points but also as physical barriers in the way of water molecules. In some cases, the HEC cross-linking led to an increase in WVP, as the cross-linking reaction may break the crystalline structure of HEC, which will increase the solubility and permeability of water [60]. Nevertheless, we used inorganic, hydrophobic particles as cross-linking points, and these formed a physical barrier for the water molecules, leading to a more tortuous pathway [33]. In addition, the presence of hydrophobic compounds from CEO also decreased the WVP value.

As the quantity of ZnO NPs from the composite films increases, the WVP values decrease as expected. Nevertheless, we can observe that even if WVP is decreasing, the values for the C3 and C4 samples are not statistically different, which indicates some sort of limiting effect from more ZnO NPs, perhaps due to local agglomeration.

### 3.7. Film Expansion Profile

**Figure 11:**
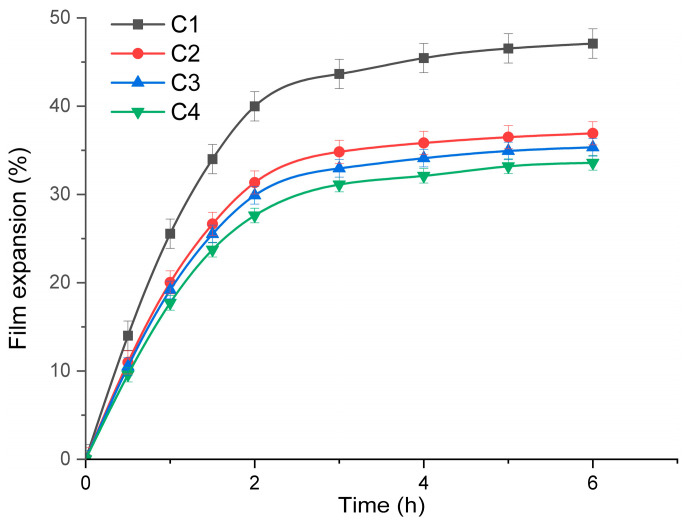
Film expansion profile in a simulated wound environment for C1–C4 samples.

For medical uses as wound dressing, the films are expected to absorb wound exudate and therefore to expand after application over a wound. The expansion profile is an important characteristic as it indicates the capacity of the films to swell by absorbing wound exudate and at the same time to diffuse/release the loaded antimicrobials [68]. The expansion profile study was made in a simulated wound environment [69], and the results are presented in Figure 11.

The films expand rapidly by absorbing moisture, the swelling rate being high in the first two hours. In the following hours, the expansion slows down, reaching values as high as 47.09% for C1, or as low as 33.58% for C4 after 6 h. All three composite films, C2–C4, present a smaller expansion percentage when compared to the simple HEC film C1. This behavior is most probably due to the presence of ZnO and SiO_2_ particles that acts as cross-linking points and hinder the movement of polymer chains, minimizing the available space for water molecules to be absorbed [64]. To sustain this hypothesis, one can observe that the expansion percentage also decreases as the ZnO NPs quantity increases, with statistical differences between the C2 and C4 samples. Excessive swelling can transform the films in free flowing gel on the wound surface or lead to the disintegration of the film [70]. One positive characteristic of the C1–C4 samples is that all films have retained their circular shapes at 6 h, without any visible disintegration.

### 3.8. Release Profile of Cinnamaldehyde

**Figure 12:**
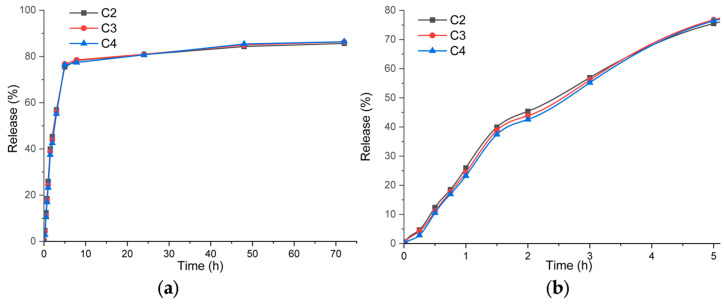
Cinnamaldehyde release profile from C2–C4 samples (**a**); detail of the release profile for the first five hours (**b**).

The release profile of cinnamaldehyde in PBS was determined by measuring the absorbance at 288 nm. The release curves shown in Figure 12 are similar, most probably due to the same CEO content loaded into the MCM-41 structure. Most of the cinnamaldehyde is released in the first five hours (~76%). After this initial burst, a slower release occurs after 72 h which releases some additional 10% cinnamaldehyde.

The detailed Figure 12b presents the release profile from the first five hours. The curves have a sigmoid shape, indicating the presence of different mechanisms. While in the first phase, the release is generated by the CEO trapped in the HEC matrix, and after 90 min, the release from the mesopores of MCM-41 becomes dominant. The minor differences among the C2–C4 samples are due to interactions with ZnO NPs that can slow the diffusion process of cinnamaldehyde. A similar slow release was reported for ferulic acid, a hydroxycinnamic acid, when encapsulated in MCM-41 [44]. On the other hand, the lack of encapsulation systems leads to a rapid release of the bioactive substances. In [71], the release of oregano essential oil from the whey protein/cellulose-based film was maximized in only 15 min.

In conclusion, the composite films are suitable as wound dressings as the initial release will be effective in blocking the infection generated by microorganisms. The following sustained release over 72 h is suitable to treat latent infections.

### 3.9. Antimicrobial Activity Assay

The capacity of the composite films to intercept and combat opportunistic pathogen contamination and colonization was further assessed against *S. aureus* and *E. coli* strains.

**Figure 13:**
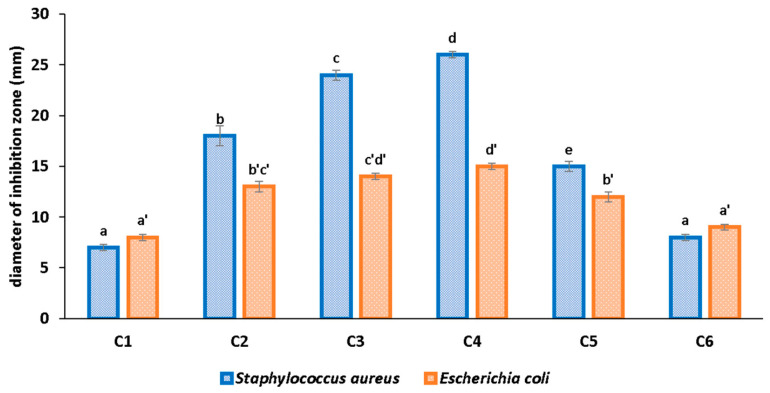
Growth inhibition diameter (mm) for C1–C4 samples, HEC-ZnO (C5) and HEC-MCM-41@CEO (C6) used for comparison; different small letters indicate statistically significant differences between films (*p* < 0.05) for each strain (a–e for *S. aureus* and a’–d’ for *E. coli*).

Some previous results indicate that HEC has a better antibacterial activity when compared with pure cellulose [5,11]. We found that the simple HEC sample has a small antimicrobial activity with a 7–8 mm growth inhibition diameter which can be also assigned to the glycerol content (Figure 13).

The growth inhibition diameter increases from the C2 to C4 samples as the ZnO quantity increases. This is more visible for the *S. aureus* strain (where the diameter of the growth inhibition zone ranges from 18 to 26 mm) than for *E. coli* (the inhibition zone increases from 13 to 15 mm), indicating a specific susceptibility of the former to the presence of ZnO NPs. For *S. aureus*, the increase of ZnO quantity leads to significant differences in growth inhibition diameter, while for *E. coli*, there are significant differences only between C and C4.

In fact, analyzing the C1, C5, and C6 antibacterial activity results, one can observe that adding MCM-41 loaded with CEO into the composite film has a minor effect, increasing marginally the growth inhibition zones, while adding ZnO into the cellulose film leads to an increase of the growth inhibition diameter by 50% for *E. coli* and more than twice for *S. aureus.* Going further by comparing C2 with C6, the addition of ZnO increases again the growth inhibition diameter by 50% for *E. coli* and more than twice for *S. aureus*. By comparing C3 with C5, we can observe that the addition of MCM-41 loaded with CEO has a modes effect in the case of *E. coli* (an increase of ~17%) but leads to a 60% increase for the *S. aureus* growth inhibition diameter. These values can indicate that *S. aureus* is significantly more susceptible to the presence of ZnO nanoparticles than *E. coli*; however, both strains are synergistic attacked by the combination of ZnO and CEO.

**Figure 14:**
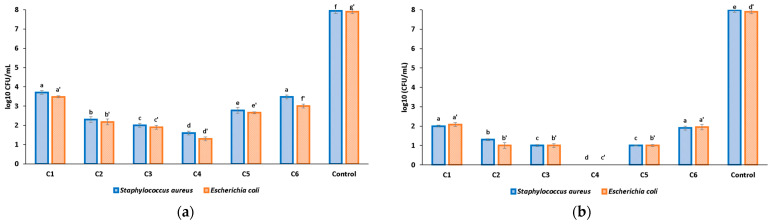
Viability of bacterial suspensions in sterile buffered saline (PBS) at 1 h (**a**) and 6 h (**b**) for C1–C4 samples; HEC-ZnO (C5) and HEC-MCM-41@CEO (C6) used for comparison; different small letters indicate statistically significant differences between films (*p* < 0.05) for each strain (a–f for *S. aureus* and a’–g’ for *E. coli*).

The viability of individual bacterial cells in direct contact with the prepared materials is important in the management and evaluation of pathogens. The results showed that all composite films present with an antibacterial effect on the free-floating cells, utilized as PBS suspensions, depending on bacterial strain and exposure time (Figure 14). The impairment of viability was visible even from the first hour of incubation, and reached a total at 24 h. The significant decrease in bacterial viability could be related to the release of bioactive species from the composite films. Inhibitory and bactericidal concentrations of bioactive species seem to be released in the culture media in the tested time frame. The mesoporous silica is a matrix that ensures the controlled and prolonged release of the CEO as seen from Section 3.8, and additionally, the presence of ZnO NPs also impairs the bacterial cells’ viability.

The C2–C4 films strongly inhibit the viability of bacterial cells even at 1 h of incubation, with the results being statistically different and the potency increasing with ZnO quantity. At 6 h, the C4 sample kills all the bacterial cells, while at 24 h, we were not able to recover any viable microbial cells from the samples encoded C2–C4. Such potent antibacterial activity promotes these novel cellulose-based composite materials for various biomedical applications, such as wound dressings or as biodegradable non-toxic food packaging [72].

**Figure 15:**
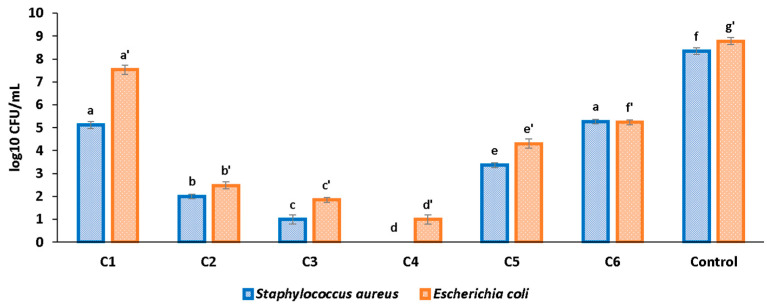
Biofilm development at 24 h for C1–C4 samples; HEC-ZnO (C5) and HEC-MCM-41@CEO (C6) used for comparison; different small letters indicate statistically significant differences between films (*p* < 0.05) for each strain (a–f for *S. aureus* and a’–g’ for *E. coli*).

To evaluate the antibiofilm effects of the cellulose-based composite films, two representative Gram-negative and Gram-positive bacterial strains (*E. coli* and *S. aureus*) were used. The data were represented as log_10_ CFU/mL (corresponding to the amount of biofilm-forming viable cells) for each sample (Figure 15).

All the tested samples exhibited inhibitory effects against *E. coli* and *S. aureus* biofilms at 24 h. It can be observed that while sample C1 containing only HEC has some impact on biofilm development, all C2–C4 composite samples have strong antibiofilm activity against both bacterial strains, with the maximum potency being developed by the C4 sample with the highest ZnO concentration. Complete inhibition of the *S. aureus* biofilm was achieved at 24 h in the case of the C4 sample, which contains the highest ZnO NP concentration and MCM-41 loaded with CEO. Sample C6 indicates that the presence of only MCM-41 loaded with CEO has a negligible effect on *S. aureus* biofilm formation but decreases the *E. coli* CFU by 2.29 log_10_ units. A stronger activity can be observed for ZnO-nanoparticle-containing samples, whose addition in C5 composite films decrease the biofilm cells’ viability by 1.75 and 3.23 logs for *S. aureus* and *E. coli*, respectively. By further comparing C2 with C6, a strong decrease by 3.26 and 2.76 logs, for *S. aureus* and *E. coli*, can be noticed at the addition of ZnO. In the case of the addition of MCM-41 loaded with CEO (comparison of C3 with C5), a decrease by 2.36 and 2.46 logs for *S. aureus* and *E. coli* can be observed. These values confirm the synergic antibacterial activity of ZnO nanoparticles and CEO. ZnO seems to have a higher impact on *S. aureus* while CEO affects preferentially *E. coli*. When both antibacterial agents are present, their effect is amplified upon total inhibition of biofilm formation. This is an important feature as the adherence of bacteria to the film surface can lead to infection establishment and the formation of complex biofilms that are hard to combat [73]. Therefore, all composite samples C2–C4 can be considered suitable for blocking the biofilm formation, the strongest activity belonging to the C4 film.

Overall, the samples are more potent against Gram-positive *S. aureus* than against Gram-negative *E. coli* strains, similar to other reports [11]. The fact that Gram-positive *S. aureus* is better inhibited than Gram-negative *E. coli* implies the existence of different antibacterial mechanisms for ZnO and CEO, and is related to the structure of the cell membrane wall. The capacity of ZnO NPs and CEO to block the biofilm formation is an important ability in developing antibacterial films containing safe, natural compounds. By comparison with traditional antibacterial substances, ZnO and CEO have the significant advantage of not being considered toxic and of not inducing bacterial resistance, thus preventing the spread of “superbugs”.

### 3.10. Biocompatibility Assessment of the Composite Materials

Biocompatibility assessment is a crucial step in the development and validation of novel materials, especially those intended for interactions with living cells. Ensuring the biocompatibility of a novel material is essential to prevent adverse effects such as cytotoxicity or inflammation, which could compromise the material’s intended application. In this context, the biocompatibility of the novel materials (C1 to C4) was evaluated using HaCaT keratinocytes by assessing cell viability and proliferation through the MTT assay, and cytotoxicity through the LDH assay.

**Figure 16:**
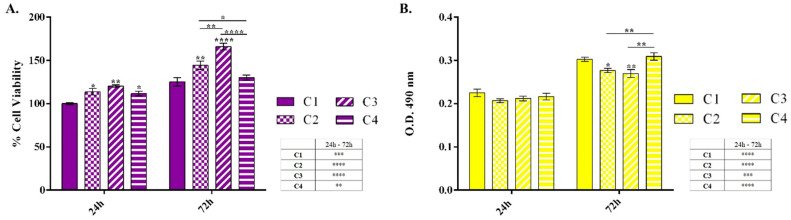
Graphical representation of (**A**) human HaCaT keratinocytes viability and proliferation potential after 24 h and 72 h of contact with the novel materials and (**B**) LDH leakage levels after 24 h and 72 h of cell–material interaction as a measure of the materials’ cytotoxic potential. Data is represented as the mean values of three independent experiments ± standard deviation. Statistical significance: * *p* ≤ 0.05; ** *p* ≤ 0.01; *** *p* ≤ 0.001; **** *p* ≤ 0.0001.

The MTT assay (Figure 16A) revealed that after 24 h of contact with the tested materials, all samples exhibited enhanced cell viability compared to the control. No significant differences in cell viability were observed between the C2, C3, and C4 formulations at this time point. However, after 72 h, both the C2 and C3 formulations induced a statistically significant increase in cell viability compared to the control, whereas cells cultured on the C4 formulation showed similar viability to the control. Among all samples, the C3 formulation demonstrated the greatest support for HaCaT metabolic activity, showing statistically significant higher cell viability compared to the C2 and C4 formulations. In terms of cell proliferation, comparing the results between the two experimental time points (24 h and 72 h) revealed that all formulations promoted HaCaT cell proliferation. However, the C2 and C3 formulations significantly enhanced cell proliferation compared to the C4 formulation, which showed a proliferation pattern similar to the control.

To assess the cytotoxic potential of the novel materials, the LDH leakage was quantified as an indicator of damage to the human keratinocyte cell membrane (Figure 16B). The results demonstrated no significant increase in LDH release after 24 h of cell contact with the materials, indicating minimal cytotoxicity across all samples. At 72 h, LDH levels remained comparable to the control, further supporting the low cytotoxicity of these materials. Notably, the C2 and C3 formulations exhibited statistically significant lower LDH leakage, suggesting that they possess the lowest cytotoxic potential among the tested samples.

Taken together, the results indicate that modifying the hydroxyethyl cellulose material (C1 formulation) improves its biocompatibility, with the C2 and C3 formulations showing superior performance in terms of supporting cell viability and proliferation while maintaining minimal cytotoxicity. The incorporation of ZnO nanoparticles, especially 0.5 g (C3 formulation), followed by 0.25 g (C2 formulation), enhances the material’s ability to promote and sustain HaCaT cell viability and proliferation. These results are likely due to the beneficial effects of ZnO in wound healing and skin regeneration processes, as well as its excellent biocompatibility [74,75,76]. However, exceeding the 0.5 g ZnO concentration did not yield additional benefits for HaCaT cell health, with results similar to the control. This may be attributed to the fact that a high concentration of ZnO is associated with toxicity, potentially inhibiting cell proliferation and even triggering apoptosis [77,78]. Therefore, it is critical to optimize ZnO concentrations during the synthesis of novel materials to avoid surpassing the threshold where ZnO transitions from being beneficial to potentially harmful.

## 4. Conclusions

Combining multiple antimicrobial agents in a single composite film is a successful strategy to obtain a strong antibacterial response by synergism. The innovative composite films were made by using non-toxic compounds like hydroxyethyl cellulose (food additive), ZnO (regarded as safe), mesoporous silica (food additive), and cinnamon essential oil (spice extract). The encapsulation of cinnamon essential oil in the pores of MCM-41 ensures a reserve of natural antimicrobial compounds that will be slowly released, thus obtaining a long-lasting antimicrobial activity.

Antibacterial efficiency against monospecific biofilms was evaluated. The individual presence of MCM-41 loaded with CEO or ZnO nanoparticles within the HEC matrix determined, at most, only moderate antibiofilm effects. Nevertheless, synergistic activity was evidenced for the composite samples containing both ZnO nanoparticles and cinnamon essential oil. The composite cellulose-based films revealed the ability for reducing or even eradicating the development of bacterial biofilms in the case of *E. coli* and *S. aureus*. The best inhibition of biofilm formation was obtained for the C4 sample with MCM-41 loaded with CEO and the highest concentration of ZnO, which led to *S. aureus* biofilm eradication at 24 h. The same sample, C4, decreased to zero the viability of both bacterial strains after 6 h. The most plausible explanation is the synergic activity between ZnO NPs and CEO. These results validated the development of a promising novel nanocomposite film for food packaging or other biomedical applications like wound dressings.

## 5. Patents

The compositions are patented under A/00336/18.06.2024.

## Figures and Tables

**Table 1:** Codification and composition of cellulose-based composite films.

Sample Code	Hydroxyethyl Cellulose (HEC)	MCM-41	Cinnamon Essential Oil (CEO)	ZnO	Glycerol
C1	5 g	-	-	-	2 mL
C2	5 g	0.25 g	1.5 mL	0.25 g	2 mL
C3	5 g	0.25 g	1.5 mL	0.50 g	2 mL
C4	5 g	0.25 g	1.5 mL	0.75 g	2 mL
C5	5 g	-	-	0.50 g	2 mL
C6	5 g	0.25 g	1.5 mL	-	2 mL

**Table 2:** Principal data from the thermal analysis of hydroxyethyl cellulose/ZnO/MCM-41@CEO composite films.

Sample	T5% (°C)	T10% (°C)	T15% (°C)	Mass Loss (%) RT-150 °C	Endo Effect-Dehydration (°C)	Mass Loss (%) 150–300 °C	Mass Loss (%) 300–600 °C	Exo Effect (°C)	Residual Mass (%)
C1	81.6	115.4	173.1	12.62%	88.3	45.54%	30.07%	774.0	1.38%
C2	100.1	141.9	171.3	11.20%	88.3	37.82%	45.34%	525.4	5.03%
C3	101.4	150.7	178.1	9.91%	83.6	36.55%	43.35%	515.1	9.91%
C4	131.4	167.8	191.5	7.27%	93.3	39.03%	41.33%	471.3	12.15%

**Table 3:** Water vapor permeability for C1–C4 film samples.

Film Code	WVP (10^−10^ g/Pa∙m∙s)
C1	0.768 ± 0.009 ^a^
C2	0.523 ± 0.015 ^b^
C3	0.446 ± 0.018 ^c^
C4	0.415 ± 0.014 ^c^

Different superscript letters indicate statistically significant differences between films (*p* < 0.05).

## Data Availability

The data presented in this study are available upon request from the corresponding authors.

## Supplementary Materials

The following supporting information can be downloaded at: https://www.mdpi.com/article/10.3390/pharmaceutics16091225/s1, Figure S1: Obtaining of the antimicrobial composite films; Figure S2: Scheme for the hydroxyethyl cellulose (HEC) based films preparation; Figure S3: The FTIR spectra for the evolved gases, extracted at 144, 233, and 313 °C from C4 thermal analysis (a); the traces for some compounds in the evolved gases CO_2_ (2355 cm^−1^), cinnamaldehyde (2733 cm^−1^), and H_2_O (3581 cm^−1^) from C4 thermal analysis (b).
