# Supplementary material for: Antimicrobial Hydroxyethyl-Cellulose-Based Composite Films with Zinc Oxide and Mesoporous Silica Loaded with Cinnamon Essential Oil
Source: Pharmaceutics. 2024 Sep 19;16(9):1225. doi: 10.3390/pharmaceutics16091225 (PMC11435203; doi:10.3390/pharmaceutics16091225)
Supplement: Supplementary file 1 [file pharmaceutics-16-01225-s001.zip › pharmaceutics-3114309-supplementary.pdf]

pharmaceutics

MDPI

Article

Antimicrobial Hydroxyethyl-Cellulose-Based Composite Films with Zinc Oxide and Mesoporous Silica Loaded with Cinnamon Essential Oil

Ludmila Motelica [1,2,3], Denisa Ficai [1,2,3], Gabriela Petrisor [1,3], Ovidiu-Cristian Oprea [1,2,3,*], Roxana-Doina Trușcă [1,2], Anton Ficai [1,2,3], Ecaterina Andronescu [1,2,3], Ariana Hudita [4] and Alina Maria Holban [2,4]

[1] Faculty of Chemical Engineering and Biotechnologies, National University of Science and Technology POLITEHNICA Bucharest, 1-7 Gh. Polizu, 011061 Bucharest, Romania
[2] National Center of Micro and Nanomaterials, National University of Science and Technology POLITEHNICA Bucharest, Splaiul Independentei 313, 060042 Bucharest, Romania
[3] Academy of Romanian Scientists, 3 Ilfov St., 050044 Bucharest, Romania
[4] Faculty of Biology, University of Bucharest, 077206 Bucharest, Romania
* Correspondence: ovidiu.oprea@upb.ro

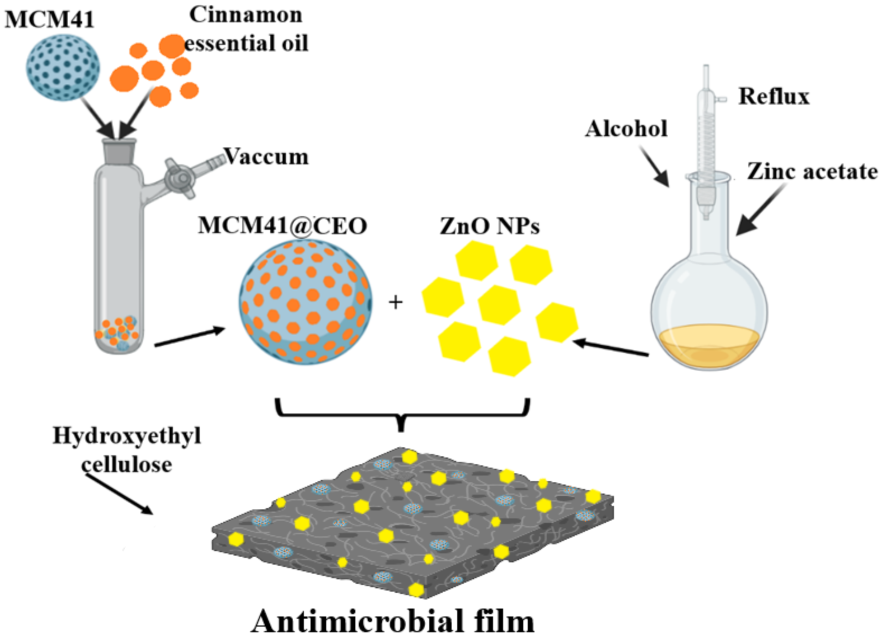

Figure S1. Obtaining of the antimicrobial composite films.

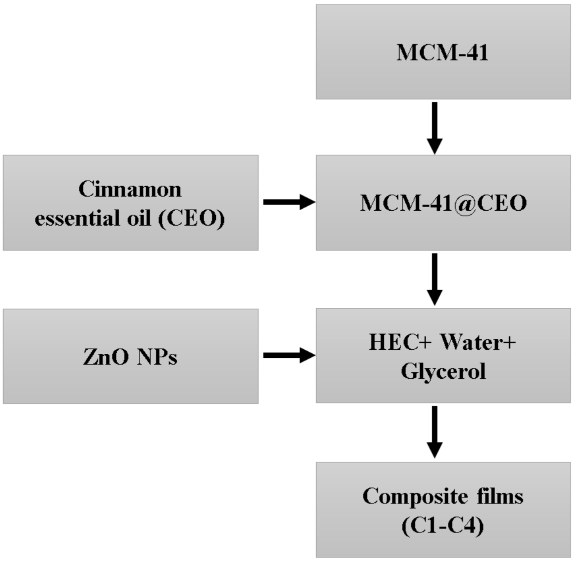

Figure S2. Scheme for the hydroxyethyl cellulose (HEC) based films preparation.

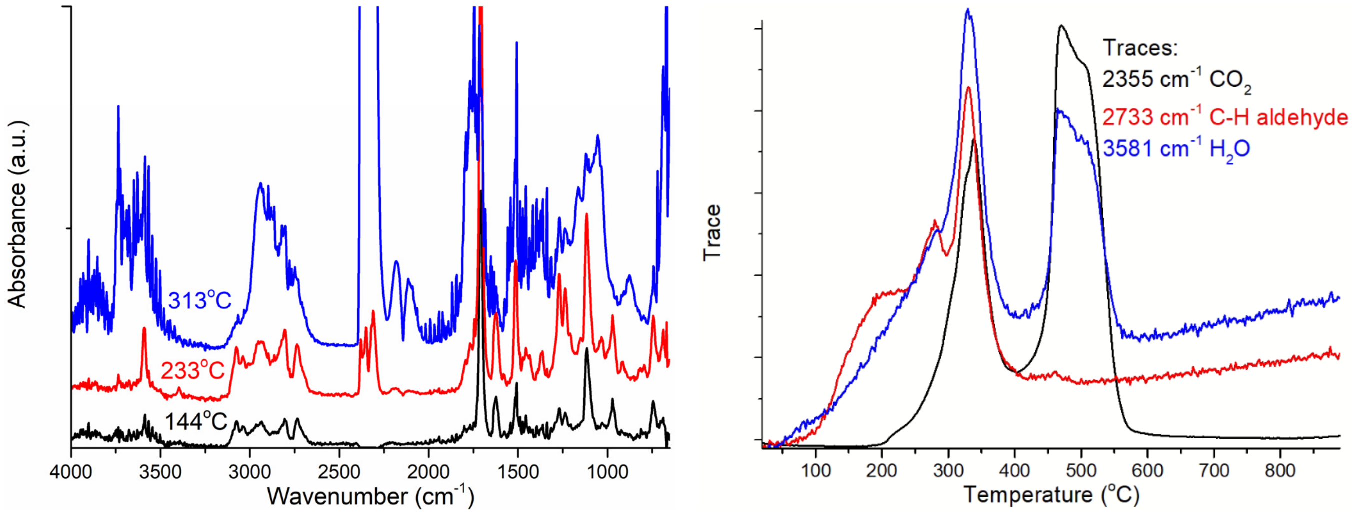

(**a**) (**b**)

Figure S3. The FTIR spectra for the evolved gases, extracted at 144, 233 and 313 °C from C4 thermal analysis (**a**); the traces for some compounds in the evolved gases CO_2 (2355 cm^{-1}), cinnamaldehyde (2733 cm^{-1}) and H_2O (3581 cm^{-1}) from C4 thermal analysis (**b**).
